# Epigenetic Regulation of Cytokine Production in Human Amnion and Villous Placenta

**DOI:** 10.1155/2012/159709

**Published:** 2012-05-14

**Authors:** Murray D. Mitchell, Anna P. Ponnampalam, Gregory E. Rice

**Affiliations:** ^1^The University of Queensland Centre for Clinical Research, Royal Brisbane Hospital, Building 71/918, Herston, QLD 4029, Australia; ^2^The Liggins Institute, University of Auckland, 2-6 Park Avenue, Private Bag 92019, Auckland 1023, New Zealand

## Abstract

The mechanisms of human preterm labour appear inextricably linked to cytokine biosynthesis by gestational tissues. In turn, cytokine production by gestational tissues has been shown to be regulated by epigenetic mechanisms. In this paper, we demonstrate that cytokine production in gestational tissues is regulated epigenetically in a tissue-specific manner. Furthermore, we show that treatment with a histone deacetylation inhibitor can partially abrogate LPS-stimulated TNF*α* production in villous placenta but not amnion. LPS treatment significantly (*P* < 0.05) increased the production of IL-1*β* (*∼*10–34-fold), TNF*α* (*∼*23–>100-fold) and IL10 (*∼*6–10-fold) after 24 h of treatment in villous explants, as expected. There were no significant LPS effects on IL1Ra production. AZA treatment did not have any significant effect on any cytokines' production tested either alone or in combination with LPS. Interestingly, however, the stimulatory effects of LPS on TNF*α* production were partially mitigated (*P* < 0.05) by TSA treatment in villous explants. We suggest caution in the consideration of histone deacetylation inhibitors in pregnancy due to the different responses in gestational tissues.

## 1. Introduction

Preterm birth is the leading cause of neonatal death worldwide with one million deaths directly resulting from premature birth. More children under the age of 5 years die due to preterm birth than to AIDS, malaria, or tuberculosis [1]. The rate of preterm birth in Australia is gradually increasing [2]. Recently, the importance of epigenetics as a regulator of gene expression has become apparent and, potentially, epigenetic differences are driving gene expression leading to preterm birth.

Epigenetics is the process by which interactions between genes and environment lead to stable changes in phenotype [3]. Epigenetic changes (e.g., DNA methylation, covalent histone modifications, etc.) are by definition heritable, meaning that effects brought about by the environment at critical periods of development (such as gestation) can have long-term detrimental consequences [4]. Indeed, certain epigenetic changes are passed between generations which might explain some of the familial and intergenerational effects observed for preterm birth [5]. Importantly and in contrast to genetic inheritance, epigenetic heritability is potentially reversible [6]. An epigenomics approach, thus, opens new avenues for therapeutic interventions.

Epigenetic information is conveyed via a synergistic interaction between mitotically heritable patterns of DNA methylation and histone-mediated modifications to the chromatin structure [7]. In mammals, DNA methylation primarily involves the addition of methyl groups to cytosine residues present in a CpG dinucleotide. Hypermethylation of promoter regions of genes is typically associated with transcriptional repression whereas hypomethylation is usually permissive for gene activity. Histone modifications consist of a plethora of enzymatic modifications including acetylation, methylation, ubiquitination, and phosphorylation of different amino acids in the N terminal tails of histone proteins, the combination of which is thought to determine the local conformational state of the chromatin. Currently, over 60 different histone modifications have been identified although actual numbers are likely to be significantly higher [8]. Histone modifications are generally considered a less stable epigenetic mark than DNA methylation. Recent work has clearly demonstrated that epigenetic marks determine time- (temporal) and tissue-specific (spatial) aspects of gene expression throughout development. In contrast, others might carry an epigenetic signature that is less sensitive to the normal stimuli of labour, therefore, leading to dysfunctional labour or postdates delivery. The environment presumably plays a crucial role in bringing about these epigenetic changes that are critical for the safe passage of gestation and later outcomes. An increasing number of diverse factors are now known to epigenetically regulate genes, including age [9], inflammation [10], gender [11], genotype [12], stress [13], nutrition [14], metabolism [15], drugs [16], and infection [17], thus heightening the relevance of the study of the epigenetics of preterm birth.

Both classes of epigenetic modification (e.g., DNA methylation and covalent histone modifications) can be reversed through treatment with a chemical inhibitor: 2′-deoxy-5-azacytidine (AZA) that inhibits DNA methylation leading to global hypomethylation of DNA, while treatment with an inhibitor of histone deacetylation such as trichostatin A (TSA) leads to increased levels of histone acetylation. In both cases, intervention favours the transcriptionally active epigenetic state.

We demonstrated in an earlier study [16] that the combination treatment of human choriodecidual explants with AZA and TSA leads to a massive increase in IL-1*β* production in response to LPS. In the present study, we extend those findings to amnion and villous placenta.

## 2. Materials and Methods

### 2.1. Reagents

Lipopolysaccharide (LPS), 5-aza-2′-deoxycytidine (AZA), and trichostatin A (TSA) were purchased from Sigma Chemical (St. Louis, MO). Culture media and fetal calf serum (FBS) were from Life Technologies (Carlsbad, CA, USA). Human IL1*β*, TNF*α*, IL10, and IL1Ra ELISA kits were purchased from BD Bio Sciences.

### 2.2. Explant Cultures

Placental tissues were collected from women undergoing elective caesarean section at term, with the approval of the NorthernX Regional Ethics Committee. Three individual placentae from singleton pregnancies of healthy nonsmoking mothers were used in the study. In triplicate, villous and amnion tissues explants were cultured in 12-well plates (three pieces of tissue to a well) in DMEM/F12 media supplemented with glutamax, 10% FBS, and 1% Antibiotic-antimycotic and incubated at 37°C in humid 5% CO_2_/95% air for 24 h (modified from [18, 19]).

Explant treatments included the use of 5-aza-2′-deoxycytidine (AZA), which inhibits DNA methylation, and/or Trichostatin A (TSA) which inhibits histone deacetylation. Doses and times were chosen to be consistent with published literature [18, 20]. 24 hours following placental dissection, the media were replaced with serum-free media (DMEM/F12 with glutamax and 0.1% Bovine gamma globulin and 1% Antibiotic-antimycotic medium). Explants were treated with either 200 nM AZA alone, TSA (300 nM) alone, 5 *μ*M AZA with TSA (300 nM), or the DMSO carrier and cultured for 48 h, with media refreshed once during this time. This represented a 48 h preincubation ± AZA (the action of AZA is passive and requires cell division for action). The treatment period commenced 72 h postdissection and consisted of the addition of 300 nM TSA or DMSO carrier in fresh media, in the presence or absence of LPS (5 *μ*g/mL), to the explants for a duration of 24 h. The media were collected following the treatment and the wet weight of the tissue in each well determined so that cytokine production rates could be normalized. Measurements of pro- (IL1*β*, TNF*α*) and anti-inflammatory (IL10, IL1Ra) cytokines were conducted on cultured media using ELISAs according to manufacturers' specifications.

### 2.3. Statistical Analysis

Production rates of cytokines were calculated as picograms per gram wet tissue weight per 24 h and are presented as % basal control values (means ± SE, *n* = 3). Statistical significance was determined by ANOVA, and *P* < 0.05 was considered to be significant.

## 3. Results

### 3.1. Effect of AZA and TSA on Cytokine Production by Villous Explants in the Presence or Absence of LPS

LPS treatment significantly (*P* < 0.05) increased the production of IL-1*β* (~10–34-fold), TNF*α* (~23–>100-fold) and IL10 (~6–10-fold) after 24 h of treatment in villous explants, as expected. There were no significant LPS effects on IL1Ra production. AZA treatment did not have any significant effect on any cytokines' production tested either alone or in combination with LPS. Interestingly, however, the stimulatory effects of LPS on TNF*α* production were partially mitigated (*P* < 0.05) by TSA treatment in villous explants.

### 3.2. Effect of AZA and TSA on Cytokine Production by Amnion explants in the Presence or Absence of LPS

Neither LPS nor the TSA or AZA had any significant effect on the cytokine production in amnion explants after 24 h of treatment.

## 4. Discussion

**Figure 1:**
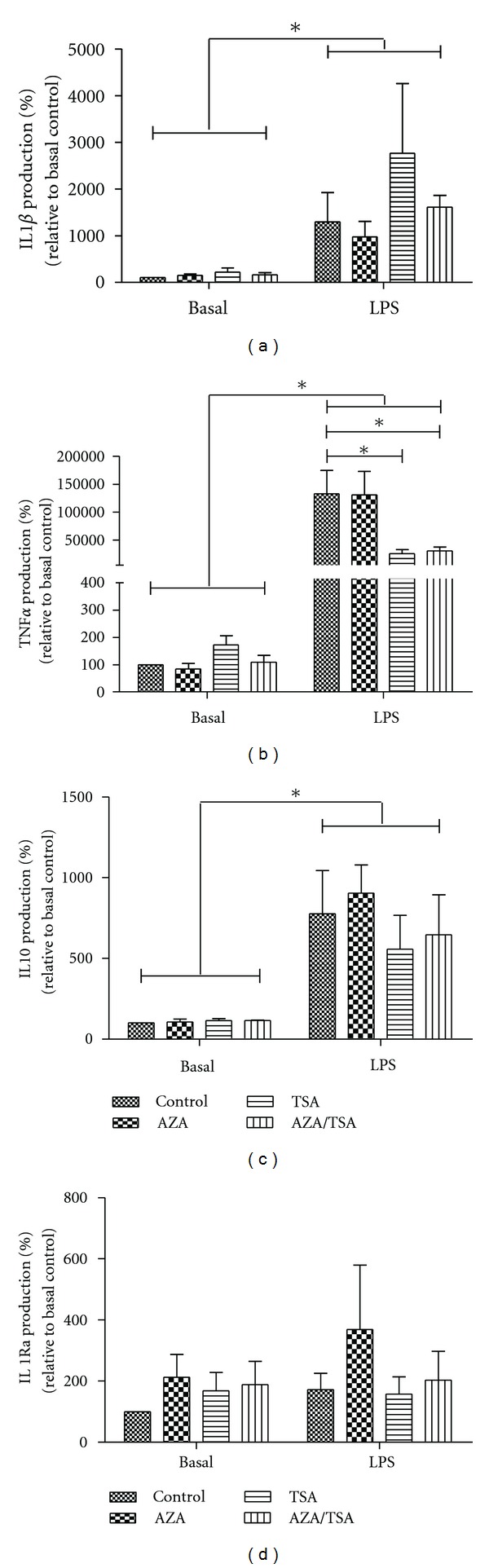
Effect of AZA and TSA on cytokine production by villous explants in the presence or absence of LPS. The levels of cytokines were measured by ELISA. The *y*-axis shows the rate of mean IL1*β* (a), TNF*α* (b), IL10 (c), and IL1Ra (d) production (as % basal control values), normalized against the wet tissue weight, and the *x*-axis shows the different treatment groups. Error bars represent 1 S.E.M. *P < 0.05, *n* = 3.

**Figure 2:**
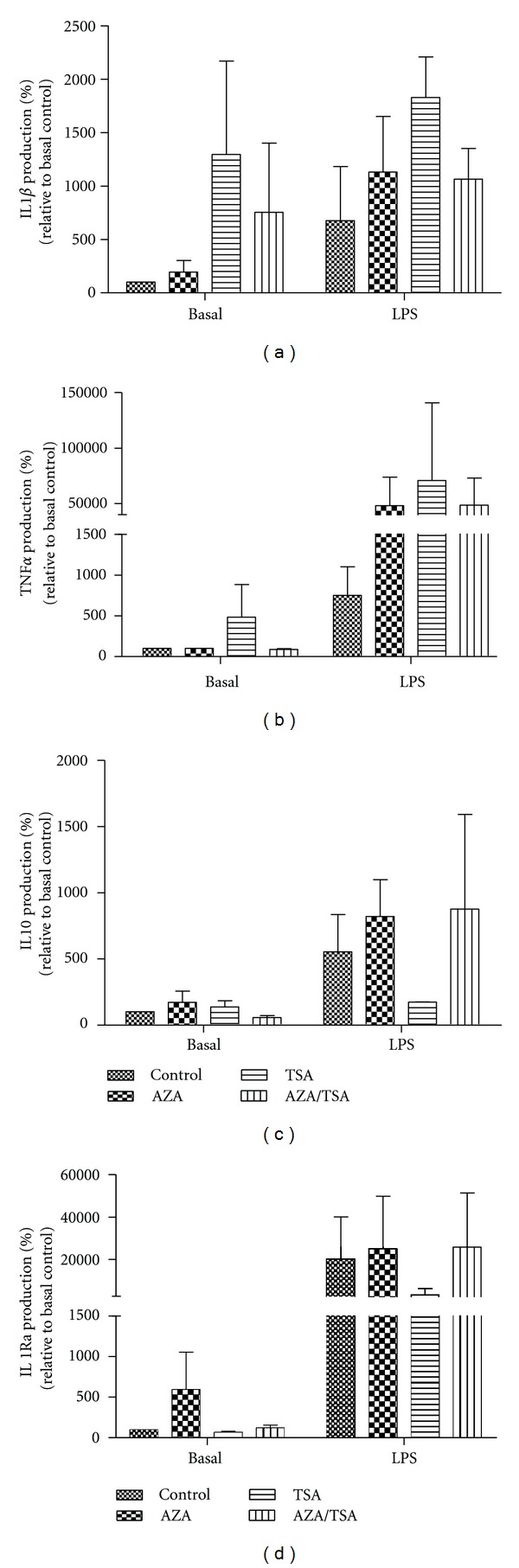
Effect of AZA and TSA on cytokine production by amnion explants in the presence or absence of LPS. The levels of cytokines were measured by ELISA. The *y*-axis shows the rate of mean IL1 *β* (a), TNF*α* (b), IL10 (c), and IL1Ra (d) production (as % basal control values), normalized against the wet tissue weight, and the *x*-axis shows the different treatment groups. Error bars represent 1 S.E.M.

Conceivably, epigenetic modifications (e.g., DNA methylation and covalent histone modifications) might render individuals more or less susceptible to either term or preterm birth by modulating the expression of genes that are effectors of the parturition pathway, for example, the contraction-associated proteins, COX-2, oxytocin, and prostaglandin receptors in myometrium [20]. Thus, some individuals may have a sensitive epigenetic phenotype that leads to an increased susceptibility to preterm labour. Global DNA methylation is increased in preterm preeclamptic placentae [21]. In this study, in conjunction with our previous work [18] we have shown that epigenetic regulation of cytokine production is tissue specific in gestational tissues. Caution of course should be exercised in the comparison of noncontemporaneous data. In choriodecidua AZA/TSA treatment resulted in a massive increase in IL-1*β* production in response to LPS whereas in amnion and villous placenta this did not occur although a small positive trend in mean production was noted (Figures 1 and 2). Our finding of TSA mitigation of LPS-stimulated TNF*α* production is most fascinating. This raises the question of TSA-like moieties being developed to attenuate inflammatory process in the human placenta. The disadvantage of such an approach is, of course, the enhancement of IL-1*β* production in choriodecidua by such an agent [18]. The tissue-specific nature of these regulatory mechanisms means that we must be cautious in our approach to such matters.

Amnion clearly does not respond to the inflammatory challenge of LPS although it remains a significant source of cytokine production and once activated can respond vigorously to modifications of the cytokine environment [22–25].

Villous placenta seems crudely to be more active in proinflammatory cytokine biosynthesis than anti-inflammatory cytokine biosynthesis. This is clearly dangerous for the fetus and may play a role in the fetal inflammatory syndrome [26], although clearly direct evidence must be obtained before conclusions are drawn.

## 5. Conclusions

Cytokine production in gestational tissues is regulated epigenetically in a tissue-specific manner. Furthermore, treatment with a histone deacetylation inhibitor can partially abrogate LPS-stimulated TNF*α* production in villous placenta but not amnion.
